# IMPLEMENTATION OF A DIGITAL COGNITIVE SCREENING PROGRAM FOR DEMENTIA IN PRIMARY CARE

**DOI:** 10.1093/geroni/igad104.1984

**Published:** 2023-12-21

**Authors:** Nicole Fowler, Judy Mullavey, Kristen Swartzell, Dustin Hammers, Jared Brosch, James Murray, Deanna Willis

**Affiliations:** Indiana University School of Medicine, Indianapolis, Indiana, United States; Linus Health, Boston, Massachusetts, United States; Indiana University School of Medicine, IU Center for Aging Research, Indianapolis, Indiana, United States; Indiana University, Indianapolis, Indiana, United States; Indiana University, Indianapolis, Indiana, United States; Davos Alzheimer’s Collaborative, Wayne, Pennsylvania, United States; Indiana University School of Medicine, Indianapolis, Indiana, United States

## Abstract

Early identification of Alzheimer’s disease and related dementias (ADRD) has become paramount given the emergence of disease modifying therapies. Integration of rapid, scalable tools to identify early cognitive impairment in primary care is essential because most at-risk individuals have limited access to specialty care. Yet, competing demands on primary care practices can make integration challenging. This demonstration project is being conducted to understand the feasibility, acceptability, and implementation of digital cognitive screening in primary care. Patients ≥65 years presenting to one of six primary care sites between 06/01/2022 and 06/30/2023 are asked to complete the Linus Health Digital Clock and Recall (DCR™) cognitive screening. Data regarding number of screening attempts that were refused, incomplete, or completed was collected. Results of the first completed screening-results were analyzed using descriptive statistics. As of 2/15/23, there are 3,920 screening attempts. DCR™ screenings were completed 40.8% of the time, refused 57.4%, and attempted but incomplete 1.8%. Thirteen-percent of attempts were positive for cognitive impairment, 37% were borderline, 44% were unimpaired, and 6% were unanalyzable. Average patient age is 73.2±6.0 years, 58% female, and 6% report less than high school education. Patients with positive screenings are older, slightly more female, and reported less education. Cognitive screening via DCR™ is ongoing and has been completed in nearly half of those approached, with half scoring cognitively impaired or borderline. This approach appears to have utility in early detection of cognitive impairment in primary care. By November 2023 we will have follow-up data for patients who screened positive.

